# An intensified trans-sectoral nutritional intervention in malnourished patients with chronic pancreatitis improves diseases prognosis and identifies potential biomarkers of nutritional status

**DOI:** 10.3389/fmed.2024.1446699

**Published:** 2024-10-08

**Authors:** Mats L. Wiese, Fabian Frost, Fatuma Meyer, Josefine Müller, Luzia Valentini, Karen Rischmüller, Georg Lamprecht, Antje Steveling, Markus M. Lerch, Ali A. Aghdassi

**Affiliations:** ^1^Department of Medicine A, University Medicine Greifswald, Greifswald, Germany; ^2^Institute of Evidence-based Dietetics (NIED), University of Applied Sciences Neubrandenburg, Neubrandenburg, Germany; ^3^Department of Medicine II, Division of Gastroenterology, Rostock University Medical Center, Rostock, Germany; ^4^LMU University Hospital, Ludwig Maximilian Universität München, Munich, Germany

**Keywords:** malnutrition, chronic pancreatitis, nutrition therapy, diet, supplementation

## Abstract

**Background:**

Malnutrition is a common complication in chronic pancreatitis and associated with reduced quality of life and life expectancy. Nutritional support is considered mandatory in malnourished patients with chronic pancreatitis but there is only scarce evidence on optimal treatment modalities and the efficacy of nutrition therapy. Here, we investigated the feasibility and efficacy of an intensified nutritional intervention in malnourished patients with chronic pancreatitis and aimed to identify suitable indicators for monitoring nutritional status.

**Methods:**

We performed a single-arm feasibility study, in which malnourished patients with chronic pancreatitis received an intensified trans-sectoral nutritional intervention for 6 months. Multimodal treatment comprised face-to-face dietary counseling, oral nutritional supplementation, and a complementary telephone-based nutrition and exercise coaching. Patients underwent follow-up examinations after 28, 90, and 180 days, when we assessed changes in anthropometric and body composition measures, muscle function, Chronic Pancreatitis Prognosis Score (COPPS), as well as blood parameters and intestinal microbiota composition.

**Results:**

Eleven out of 73 patients initially screened for study participation were enrolled in the trial of which 9 subjects (age (mean ± SD): 56.2 (±14.8) years; male: 67%; alcoholic etiology: 44%) underwent the complete intervention. Patients gained a median of 5.3 kg (8.6%) body weight, including 1.6 kg skeletal muscle mass, and significantly increased gait speed (*p* < 0.001). Ameliorated nutritional status and muscle function were associated with increased blood levels of IGF-1 and cholinesterase as well as altered gut microbiota composition on the phyla and genera level. Moreover, significant improvements in COPPS indicated reduced disease severity after 90 and 180 days.

**Conclusion:**

Malnourished patients with chronic pancreatitis benefit from intensified nutritional therapy. Besides ameliorated nutritional status, a multimodal intervention can improve muscle function as well disease prognosis. Future studies are needed to prove superiority to standard-of-care and to validate potential biomarkers for prospective monitoring of nutritional status.

**Clinical trial registration:**

https://clinicaltrials.gov/study/NCT04476056, NCT04476056.

## 1 Introduction

Chronic pancreatitis is a progressive fibro-inflammatory disease with an annual incidence of approximately 10 cases per 100,000 inhabitants (1–3). Chronic pancreatitis causes pain and eventually leads to the irreversible loss of exocrine and endocrine organ function. Due to these pathophysiological changes, patients with chronic pancreatitis have a high risk of malnutrition, which exacerbates a decline in quality of life and life expectancy (4, 5). Recent data suggest that half or more patients with chronic pancreatitis may be affected by malnutrition and a characteristic loss of skeletal muscle mass (6, 7). There is consensus that nutritional therapy is indicated in malnourished patients with chronic pancreatitis but little is known about the optimal modalities of treatment (8, 9). Well-designed, prospective studies showing the efficacy of nutritional therapy are scarce. Therefore, most nutritional recommendations are based on low evidence, which ultimately hampers implementation in clinical practice. Moreover, there is still uncertainty regarding adequate, easily accessible parameters to monitor nutritional status of malnourished patients with chronic pancreatitis (10).

To address these gaps in knowledge, we investigated the feasibility and efficacy of an intensified nutritional intervention in malnourished patients with chronic pancreatitis and studied anthropometric and biochemical changes to identify suitable parameters for monitoring nutritional status.

## 2 Materials and methods

### 2.1 Study design and population

This trial was designed as a multicenter, single-arm feasibility study conducted between May 2019 and September 2021 in the state of Mecklenburg-Vorpommern, located in northeast Germany. At University Medicine Greifswald, a local tertiary referral center, we enrolled in- and out-patients 18 years or older with confirmed diagnosis of chronic pancreatitis and concomitant malnutrition for an intensified trans-sectoral nutritional intervention carried out in collaboration with University of Applied Sciences Neubrandenburg. Diagnosis of chronic pancreatitis was based on characteristic findings in imaging studies, including endoscopic ultrasound, computed tomography, or magnetic resonance imaging with magnetic resonance cholangiopancreatography, and/or histology. The international consensus criteria by the Global Leadership Initiative on Malnutrition (GLIM) were applied to diagnose malnutrition (11). Details on the diagnostic approach including methods and cut-offs employed for patients with chronic pancreatitis have been reported previously (6). Exclusion criteria were as follows: (1) diagnosis of any malignant disease within the past 3 years, (2) pregnancy or lactation, (3) concomitant other severe chronic gastrointestinal disease, including liver cirrhosis, or (4) relevant cognitive and/or physical restraints.

The study was approved by the Institutional Review Board at the University Medicine Greifswald (internal registration number: BB 069/19) and registered at clinicaltrials.gov (NCT04476056). The intervention and all other study-related procedures were conducted in accordance with the ethical principles related to the Declaration of Helsinki.

### 2.2 Intervention

**Figure 1:**
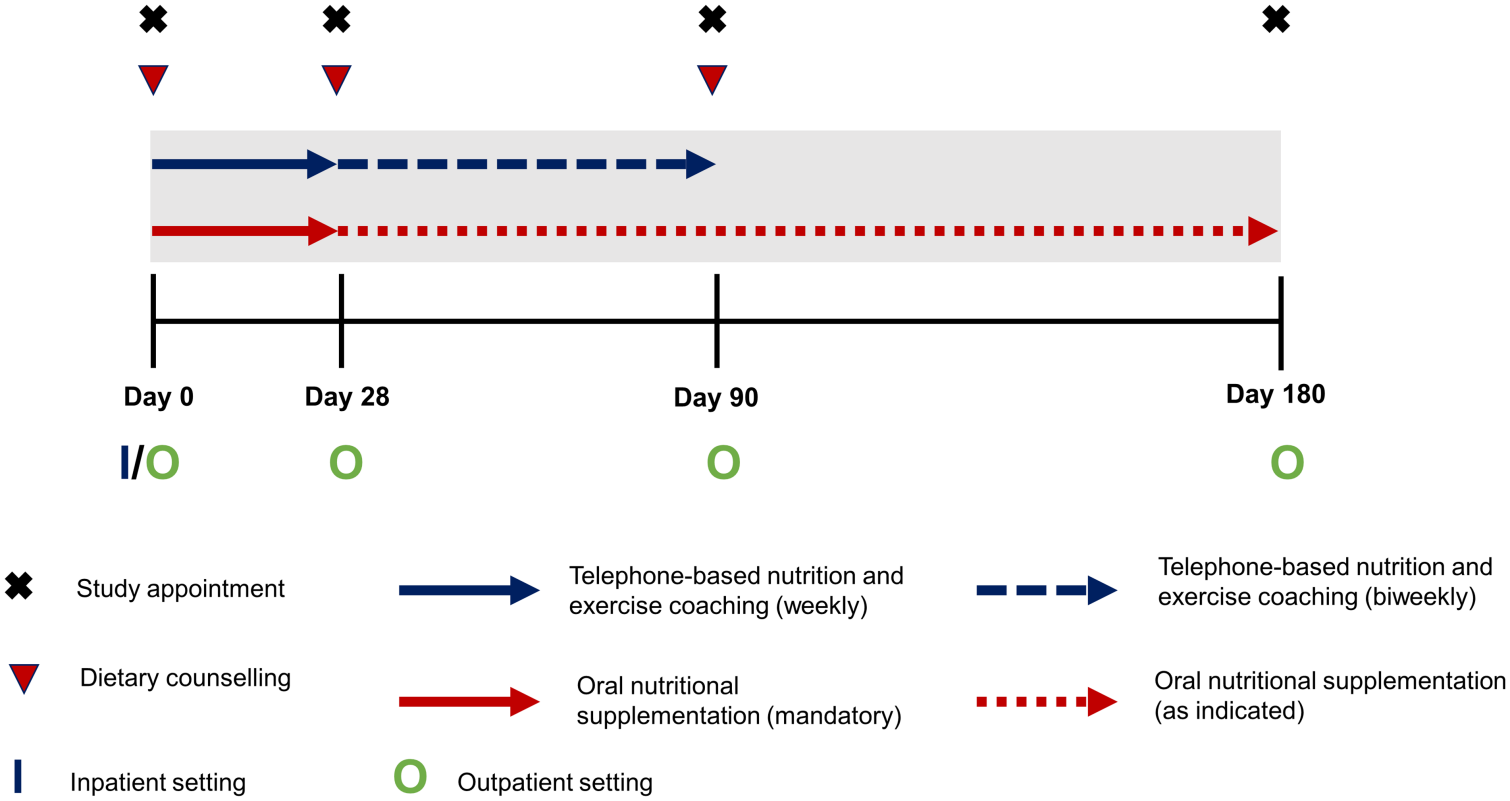
Schematic outline of the intensified trans-sectoral nutritional intervention for the treatment of malnourished patients with chronic pancreatitis.

The trans-sectoral nutritional intervention lasted over 6 months and was based on a multimodal approach comprising face-to-face dietary counseling, supplementation with oral nutritional supplements (ONS), as well as a complementary telephone-based nutrition and exercise coaching (Figure 1). Dietary counseling and complementary coaching were carried out in a standardized way according to the German Nutrition Care Process (12) by trained dietitians or nutritionists. Both in- and outpatients received their first session of dietary counseling upon study enrollment, followed by two additional sessions in an outpatient setting after 28 and 90 days, respectively. During these sessions patients were advised on an energy adequate, high protein diet (> 1.5 g/kg bodyweight) respecting individual food tolerance as well as appropriate pancreatic enzyme replacement therapy in case of exocrine pancreatic insufficiency. In addition, alcohol and smoking abstinence were promoted during the counseling sessions. Implementation of these recommendations was supported during the complementary telephone-based nutrition coaching, which took place during the first 90 days of the intervention and was administered by qualified health care professionals at University of Applied Sciences Neubrandenburg. The exercise component of coaching promoted increased everyday physical activity and exercises appropriate to the patients’ performance status, e.g., light resistance band training. During the first 28 days, phone calls were scheduled weekly and then biweekly thereafter. Regarding supplementation, for the first 28 days patients were instructed to consume daily at least two bottles of a commercial ONS (Fortimel Compact 2.4, Nutricia) containing 300 kcal and 12 g of protein per serving and to record their actual intake. After that, patients were free to continue supplementation, as indicated to achieve adequate energy and nutrient intake, throughout the intervention but without any preset targets regarding quantity of ONS.

### 2.3 Clinical and patient data

We collected personal and disease-related characteristics by standardized interview or from the patient files. Chronic Pancreatitis Prognosis Score (COPPS), a validated scoring system to predict short-term prognosis in patients with chronic pancreatitis, was used to evaluate disease severity (13). Exocrine pancreatic function was assessed by measurement of fecal elastase using a monospecific enzyme-linked immunosorbent assay (R-Biopharm AG, Darmstadt, Germany) with values of 200 μg/g or less defining exocrine insufficiency. Diagnosis of pancreatogenic diabetes was made based on the presence of major and minor criteria as suggested by Ewald and Bretzel (14). We inquired patients’ dietary intake, excluding ONS, by a validated semi-quantitative food frequency questionnaire (15) and employed the short form of the International Physical Activity Questionnaire to assess physical activity.

### 2.4 Physical examination and blood testing

At all study visits, we performed the following anthropometric measurements in patients using a standardized protocol including repeated measurements: body weight and height, waist, hip and mid-upper arm circumference as well as triceps skinfold thickness. To minimize intra- and inter-operator error, all investigators received comprehensive training based on this protocol. Adequate operator performance was ascertained before the beginning of the study. We further analyzed patients’ body composition with an eight-electrode, phase-sensitive, segmental bioelectrical impedance analysis device (mBCA 515, seca, Hamburg, Germany). For assessment of muscle strength, we tested handgrip strength of patients with the Jamar Plus+ Digital Hand Dynamometer (Patterson Medical, Warrenville, IL, United States) and carried out the 4-m gait speed test to assess muscle performance. Blood of patients was drawn to measure routine laboratory parameters markers associated with inflammation or nutritional status.

### 2.5 Microbiome analyses

We performed 16S rRNA gene sequencing of DNA extracted from fecal samples provided by the patients. Sample material was collected by the patients at home or in hospital and preserved in a test tube, which contained stabilizing DNA buffer. Following extraction (PSP Spin Stool DNA Kit; Stratec Biomedical AG, Birkenfeld, Germany), the DNA was stored at −20°C until analysis by 16S rRNA gene sequencing of the V1–V2 region, which was performed on a MiSeq platform (Illumina, San Diego, California, USA). For data processing and taxonomy assignment, we employed the open-source software package DADA2 (v.1.10) (16). For the analysis, samples were normalized to 10,000 16S rRNA gene read counts.

### 2.6 Statistical analysis

Continuous variables are presented as mean (±SD) or median (IQR) for normally and non-normally distributed data, respectively. Categorical variables are given as absolute and relative frequency. Significance of changes in parameters during the intervention were tested by Friedman test, given non-normal distribution of the data. In case of significant differences by Friedman test, we performed post-hoc analysis using the Conover test with false detection rate correction. Gut microbiome changes were analyzed between composition at Day 0 and Day 180 at the phyla and genera level. For this, we compared all phyla and genera present in more than 50% of samples using the Wilcoxon signed-rank test. Correlations between changes in taxonomic units and parameters of body composition and muscle function were then tested by calculation of Spearmen correlation coefficients. All microbiome analyses were performed on data of 7 individuals as two patients did not provide stool samples at Day 180. All results are presented on per protocol basis. Intention-to-treat analyses were performed, where applicable, and yielded comparable results. A *p*-value <0.05 was defined as statistically significant. All statistical analyses and graphical visualization were performed using R software (R Core Team, Vienna, Austria) for statistical computing (version 4.1.0). In figures in which we report data for single individuals, colored dots represent the same patient throughout the manuscript.

## 3 Results

### 3.1 Patient selection and characteristics

**Figure 2:**
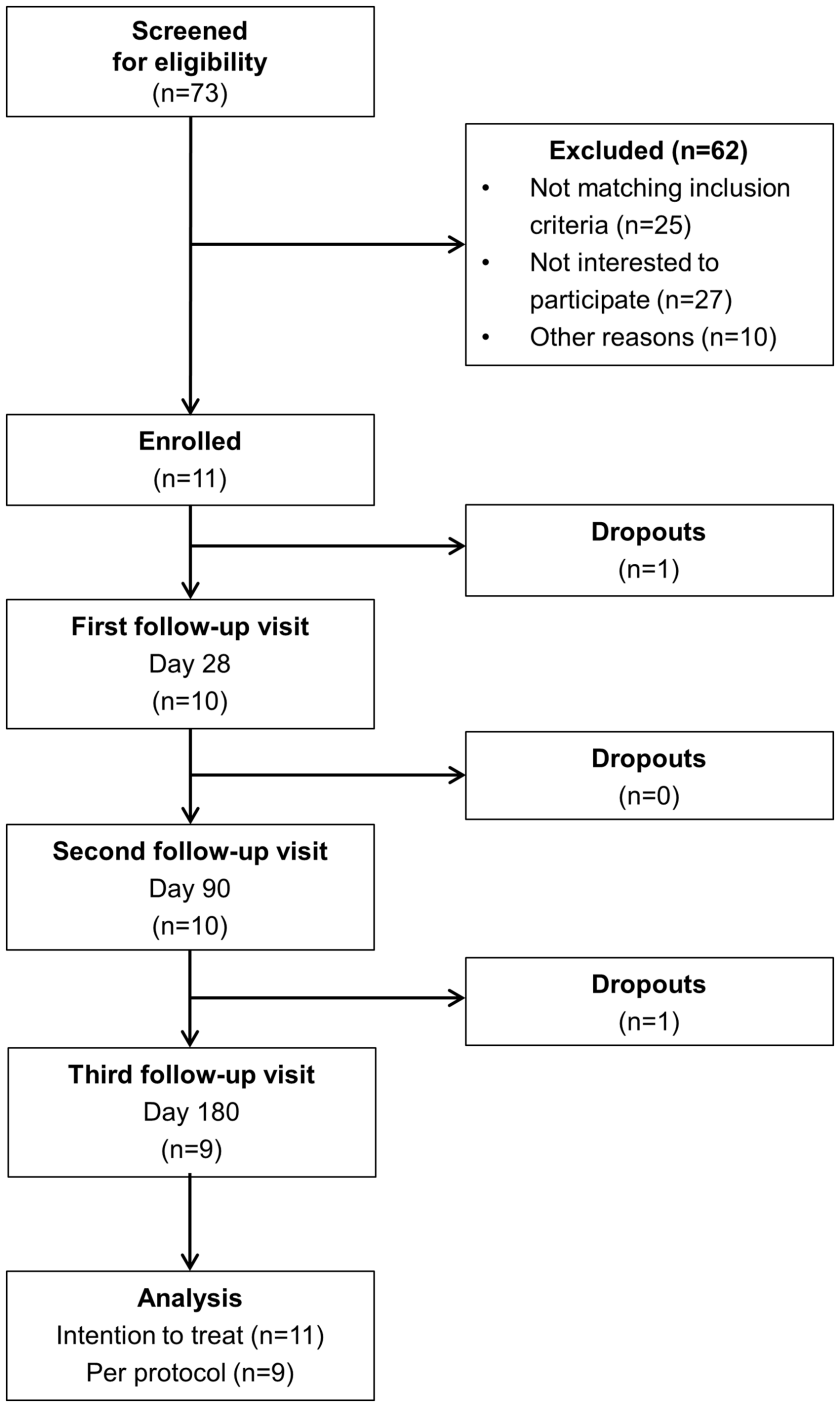
Flowchart illustrating the process of patient selection.

The process of patient selection is illustrated in Figure 2. Out of 73 initially screened patients with chronic pancreatitis, 11 individuals were eventually enrolled in the study. Among the 62 excluded patients, lack of interest to participate (*n* = 27) and non-fulfillment of inclusion criteria (*n* = 25) were the most common reasons for exclusion. Nine subjects completed the entire 6-months intervention. One patient dropped out before the first scheduled follow-up visit and another one after the second visit.

Relevant demographic and clinical patient characteristics are summarized in Table 1. Mean age of patients was 56.2 years and two thirds were male. Most patients (78%) were recruited as outpatients. The etiology of chronic pancreatitis was either alcoholic (56%) or idiopathic (44%). At study enrollment, alcohol abuse persisted in 22% of individuals and 67% continued smoking. Two thirds of patients had exocrine pancreatic insufficiency based on fecal elastase test. Yet, all but one subject already received pancreatic enzyme replacement therapy at baseline. Pancreatogenic diabetes was present in 33% of patients, who all had all undergone pancreatic surgery before. Time after surgery ranged from 2 to 31 months. Around half of the subjects had received diagnosis of chronic pancreatitis within the last 12 months and 22% required permanent opioid treatment, which was continued unchanged throughout the intervention. Disease severity was equally distributed among grades of COPPS. All patients reported unintentional weight loss, with a median body weight decrease of 7.7 and 19.9% in the last 6 and 24 months, respectively. With one exception all subjects were diagnosed as severely malnourished according to the GLIM criteria.

**Table 1:** Demographic and clinical characteristics of malnourished patients with chronic pancreatitis participating in the intensified trans-sectoral nutritional intervention (*n* = 9).

Age, yrs^a^	56.2 (±14.8)
Male sex, *n* (%)	6 (67)
Outpatients, *n* (%)	7 (78)
Etiology, *n* (%)	
Alcohol	5 (56)
Idiopathic	4 (44)
Continued substance abuse, *n* (%)	
Alcohol	2 (22)
Smoking	6 (67)
Exocrine pancreatic insufficiency, *n* (%)	6 (67)
Pancreatic enzyme replacement therapy, *n* (%)	8 (89)
Endocrine pancreatic insufficiency, *n* (%)	3 (33)
Pancreatic surgery, *n* (%)	3 (33)
Opioid treatment, *n* (%)	2 (22)
NRS of pain (0–10)^b^	2 (6)
Diagnosis of chronic pancreatitis within past 12 months, *n* (%)	55 (56)
COPPS, *n* (%)	
A	3 (33)
B	3 (33)
C	3 (33)
Weight loss, %^b^	
Past 6 months	7.7 (15.5)
Past 24 months	19.9 (16.8)
Grade of malnutrition^c^, *n* (%)	
Moderate	1 (11)
Severe	8 (89)

COPPS, Chronic Pancreatitis Prognosis Score; NRS of pain, Numeric Rating Scale of Pain: 0 = no pain, 10 = worst possible pain (past 7 days). ^a^Value is presented as mean (±SD). ^b^Values are presented as median (IQR). ^c^Diagnosis based on the Global Leadership Initiative on Malnutrition criteria.

### 3.2 Compliance

**Figure 3:**
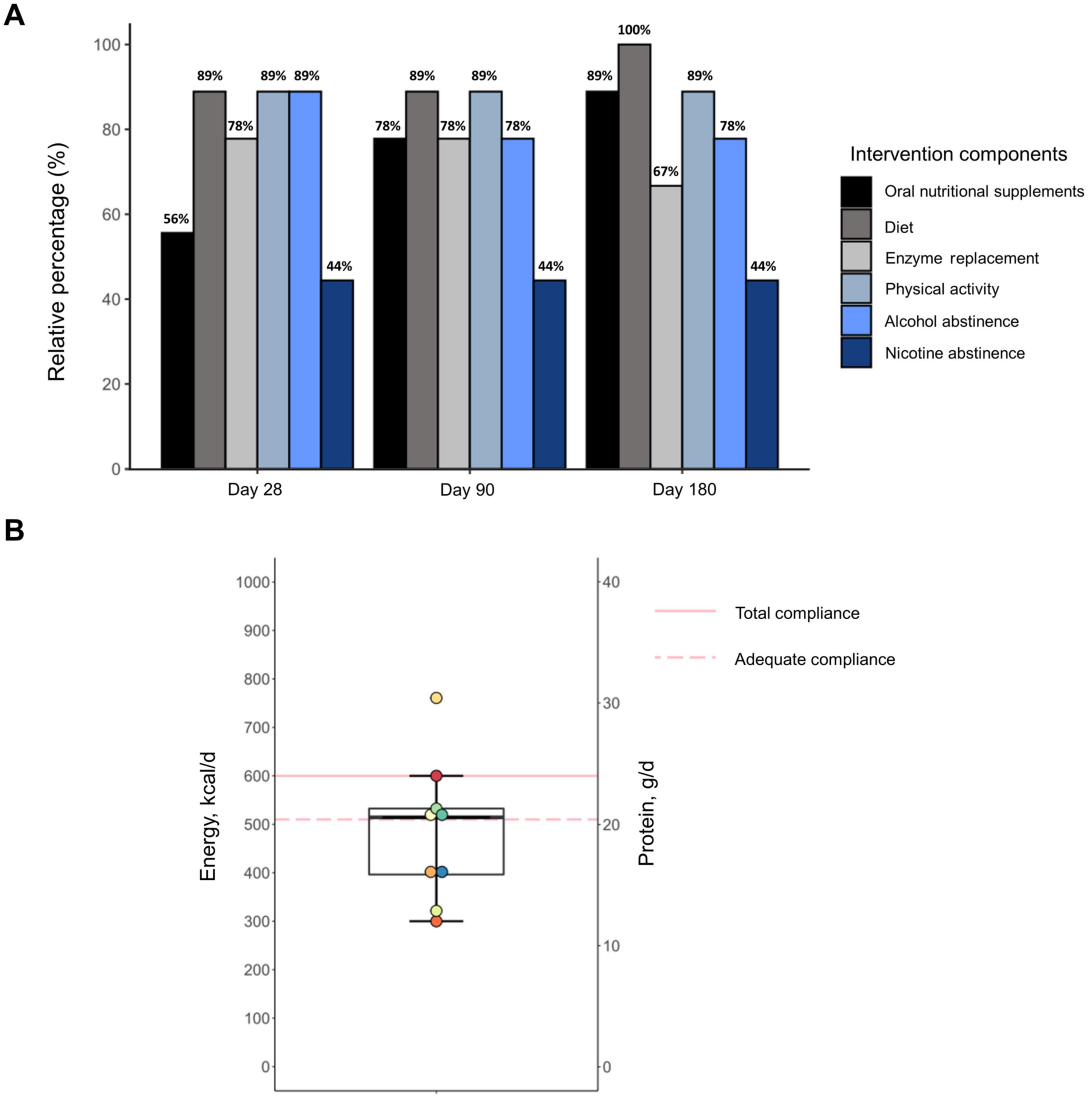
Adherence of malnourished patients with chronic pancreatitis to individual components of the intensified trans-sectoral nutritional intervention **(A)** and consumption of oral nutritional supplements in the first 28 days **(B)** (*n* = 9).

Most patients showed good adherence to all individual intervention components during the entire study period (Figure 3A). Smoking abstinence was the only recommendation to which less than half of patients (44%) adhered. Notably, 33% of subjects had already ceased smoking before study enrollment and smoking behavior did not change during the intervention in any individual. In contrast, compliance to ONS improved over the study course. In the first 28 days, 56% of patients showed compliance, which was defined as consuming on overage at least two bottles on 6 out of 7 days a week. Based on a consumption record conducted by the patients, ONS provided a median energy intake of 514 kcal and 21 g of protein (Figure 3B). For the remaining study period, when ONS quantity was based on individual requirements, compliance increased to 78 and 89% at days 90 and 180, respectively. All subjects required ONS at least until Day 90 and 56% continued consumption during the entire intervention. Although patients showed high adherence to dietary counseling, intakes of energy and macronutrients from oral food remained unchanged during the intervention (Supplementary Table S1). Moreover, patients were highly compliant to the exercise coaching resulting in increased physical activity which was maintained throughout the entire study (Supplementary Table S2).

### 3.3 Changes in anthropometric, body composition, and muscle function parameters

The 6-months intervention led to significant changes in most anthropometric and body composition parameters (Table 2). All but one patient, who had edema and ascites at study enrollment, gained body weight during the intervention. Body weight increased by a median of 5.3 kg (8.6%) of which 3.5 kg were fat mass and 1.6 kg skeletal muscle mass. Post-hoc analyses revealed that no significant changes could be observed after 28 days but only after 90 and 180 days of intervention.

**Table 2:** Changes in anthropometric and body composition parameters of malnourished patients with chronic pancreatitis in the course of the intensified trans-sectoral nutritional intervention (*n* = 9).

	Day 0	Day 28	Day 90	Day 180	*p*-value^1^
Body mass index, kg/m^2^	19.9 (4.0)	20.1 (4.4)	21.6 (3.97)^#^^,^^†^	22.1 (6.1)^#^^,^^†^^,^^‡^	**0.001**
Waist circumference, cm	79.4 (15.0)	83.4 (11.6)	84.9 (7.8)^#^	88.7 (12.0)^#^^,^^†^^,^^‡^	**0.007**
Hip circumference, cm	86.8 (8.2)	88.8 (8.3)	92.4 (11.6)^#^^,^^†^	91.8 (13.8)^#^^,^^†^^,^^‡^	**0.003**
Waist-to-Hip ratio	0.90 (0.11)	0.92 (0.15)	0.92 (0.11)	0.93 (0.12)	0.177
Mid upper arm circumference, cm	24.7 (5.5)	24.2 (5.3)	25.4 (4.4)^#^^,^^†^	25.4 (5.0)^#^^,^^†^^,^^‡^	**<0.001**
Triceps skinfold thickness, mm	13.4 (8.4)	14.6 (13.0)	13.9 (6.7)	14.0 (7.8)	0.072
Fat mass index, kg/m^2^	5.1 (2.7)	5.5 (2.5)	5.7 (2.8)^#^^,^^†^	6.6 (4.1)^#^^,^^†^^,^^‡^	**0.002**
Fat free mass index, kg/m^2^	16.6 (4.3)	16.1 (3.6)	16.6 (3.7)	16.3 (3.3)	0.057
Skeletal muscle mass index, kg/m^2^	6.2 (1.8)	6.8 (1.5)	7.5 (1.8)^#^^,^^†^	7.4 (1.9)^#^^,^^†^	**0.001**
Skeletal muscle to fat mass ratio	1.31 (0.56)	1.26 (0.75)	1.19 (0.79)	1.10 (0.070)	0.131
Phase angle, °	4.2 (1.1)	4.3 (0.4)	4.2 (1.0)	4.4 (0.9)^#^^,^^†^^,^^‡^	**0.011**
Total body water, L	34.5 (7.7)	33.8 (7.0)	35.0 (7.1)^#^	35.2 (6.9)^#^^,^^†^	**0.015**
Extracellular body water, L	14.9 (4.0)	15.1 (3.2)	14.9 (2.7)	14.9 (2.4)	0.725
Extracellular to total body water ratio	0.466 (0.057)	0.458 (0.029)	0.458 (0.043)	0.456 (0.047)	0.095

All data is presented as median (IQR).

1 Changes over time were tested using Friedman test.

# Indicates significant difference from Day 0 based on Conover post-hoc test with correction for false detection rate, *p* < 0.05.

† Indicates significant difference from Day 28 based on Conover post-hoc test with correction for false detection rate, *p* < 0.05.

‡ Indicates significant difference from Day 90 based on Conover post-hoc test with correction for false detection rate, *p* < 0.05.Bold values denote statistical significance at the *p* < 0.05 level.

**Figure 4:**
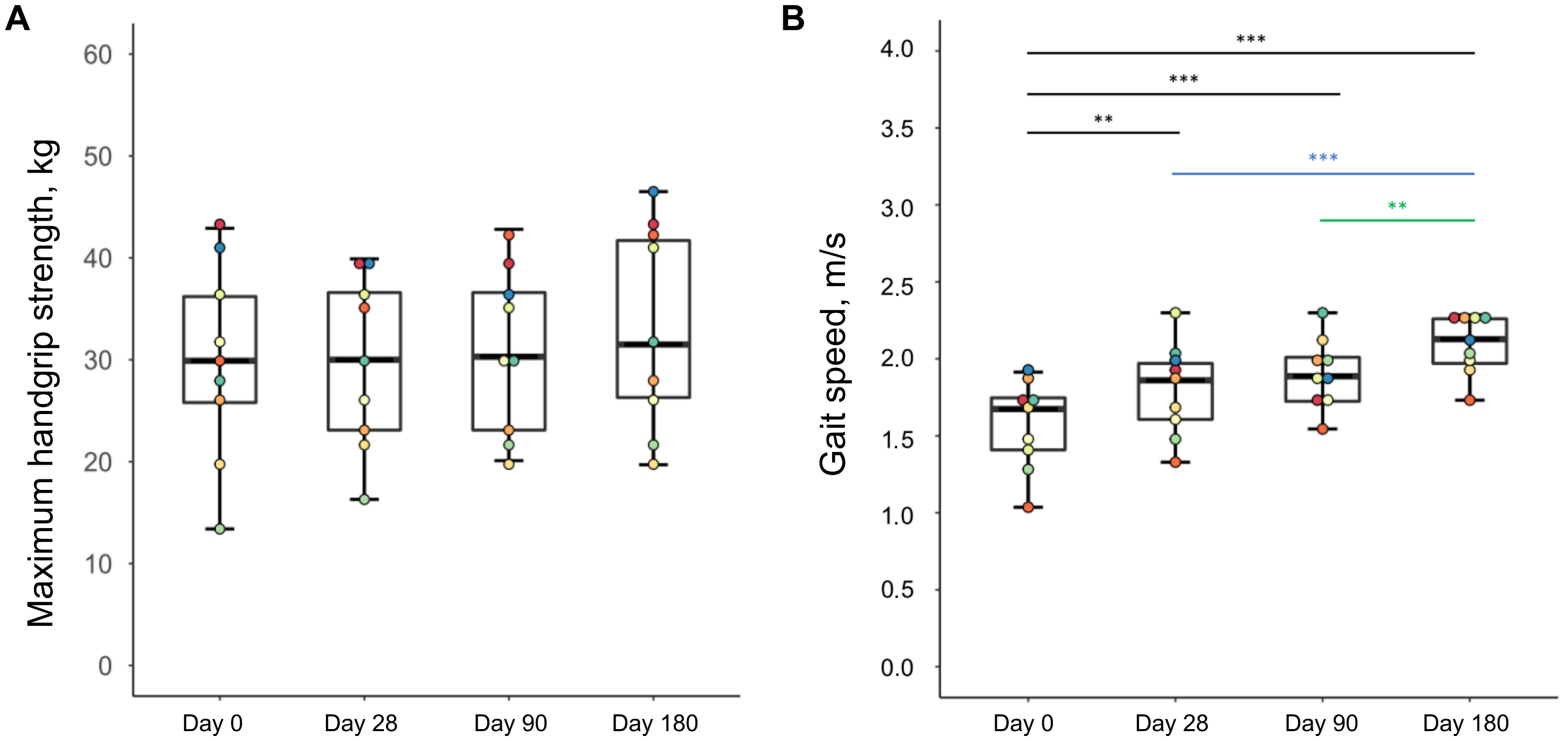
Changes in handgrip strength **(A)** and gait speed **(B)** of malnourished patients with chronic pancreatitis in the course of the intensified trans-sectoral nutritional intervention (*n* = 9). **p < 0.01; ***p < 0.001.

Besides the gains in skeletal muscles mass, there were also changes in muscle function (Figure 4). While maximum handgrip strength did not improve significantly during the intervention (*p* = 0.152), there was a continuous increase in gait speed until the study end examination (*p* = 0.001).

### 3.4 Changes in disease severity

**Figure 5:**
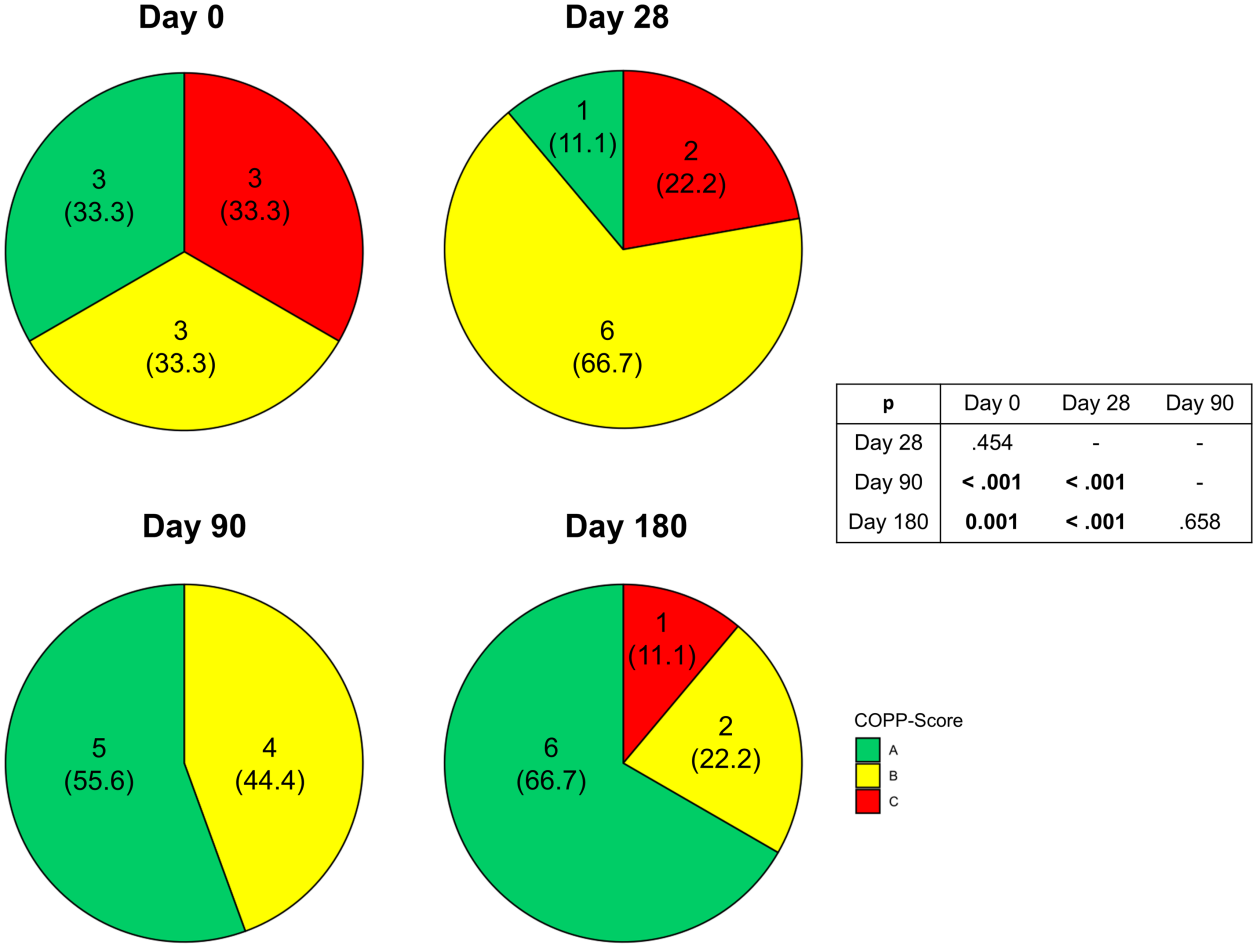
Changes in Chronic Pancreatitis Prognosis Score (COPPS) of malnourished patients with chronic pancreatitis in the course of the intensified trans-sectoral nutritional intervention (*n* = 9).

During the intervention we observed a significant amelioration of disease severity as indicated by COPPS (*p* = 0.006) (Figure 5). While at enrollment there was an equal distribution between the three grades of COPPS, at the end of intervention two-thirds of patients had an A score. Changes in disease severity could not be detected before Day 90. In the following 3 months improved COPPS status was maintained until the end of the study. Regarding individual parameters of COPPS, especially body mass index improved whereas pain scores remained unchanged (Supplementary Table S3). However, even when omitting body mass index from the calculation there was still a significant improvement in the total score (*p* = 0.049).

### 3.5 Changes in blood parameters and gut microbiota composition

Most of the blood parameters that were analyzed remained unaltered during the intervention period (Table 3). However, we detected significant changes in three parameters, i.e., cholinesterase, IGF-1, and HDL cholesterol. For each of these markers, there was a significant rise after 90 days compared to the baseline measurement. Notably, cholinesterase was the only parameter that continuously increased over 6 months whereas IGF-1 remained stable after 90 days and HDL cholesterol levels declined.

**Table 3:** Changes in blood parameters of malnourished patients with chronic pancreatitis in the course of the intensified trans-sectoral nutritional intervention (*n* = 9).

	Reference range	Day 0	Day 28	Day 90	Day 180	*p*-value^1^
Complete blood count						
Hemoglobin, mmol/L	7.4–11.2	7.6 (1.5)	7.8 (1.2)	8.1 (1.7)	8.3 (1.5)	0.087
Hematocrit, L/L	0.350–0.510	0.369 (0.085)	0.381 (0.077)	0.393 (0.074)	0.407 (0.076)	0.091
Mean corpuscular volume, fL	80.0–95.0	88.7 (5.8)	89.9 (2.6)	89.3 (4.6)	90.3 (5.4)	0.577
Mean corpuscular hematocrit, fmol	1.68–2.00	1.85 (0.11)	1.85 (0.10)	1.86 (0.11)	1.86 (0.10)	0.427
Mean corpuscular hemoglobin concentration, mmol/L	18.5–22.5	20.5 (1.4)	20.4 (0.7)	20.4 (1.0)	20.5 (0.7)	0.462
White blood cell count, 10^9^/L	4.30–10.00	7.46 (6.80)	6.74 (3.70)	7.43 (4.20)	7.82 (5.90)	0.081
Platelet count, 10^9^/L	140–440	219 (150)	267 (215)	254 (115)	249 (118)	0.145
Blood chemistry						
Creatinine, μmol/L	42–97	67 (48)	77 (32)	72 (28)	65 (32)	0.945
Alanine aminotransferase, IU/L	< 46.2	28.2 (17.4)	28.2 (15.3)	31.2 (12.9)	25.8 (5.7)	0.100
Aspartate aminotransferase, IU/L	< 35.4	20.4 (8.7)	19.2 (13.2)	20.4 (10.2)	18.0 (6.0)	0.392
Gamma-glutamyl transferase, IU/L	0.00–57.6	52.2 (100.2)	84.0 (97.5)	84.0 (125.9)	72.0 (63.3)	0.154
Alkaline phosphatase, IU/L	49.8–135.6	78.0 (111.0)	96.0 (57.0)	90.0 (45.0)	102.0 (39.0)	0.653
Cholinesterase, kU/L	5.9–19.0	8.3 (4.1)	11.7 (3.1)^#^	11.5 (4.6)^#^	12.4 (5.0)^#^^,^^†^^,^^‡^	**< 0.001**
Total bilirubin, μmol/L	0.0–17.0	6.9 (5.6)	7.8 (3.6)	5.0 (2.6)	6.8 (2.7)	0.082
Blood urea nitrogen, mmol/L	2.5–6.4	4.8 (3.4)	4.5 (3.8)	6.2 (3.2)	4.8 (3.3)	0.189
Uric acid, μmol/L	155–428	303 (228)	314 (102)	316 (98)	314 (136)	0.954
Iron, μmol/L	7.0–30.0	14.0 (8.1)	16.0 (5.5)	16.0 (8.1)	15.0 (9.7)	0.105
Albumin, g/L	34–50	37 (15)	37 (7)	38 (4)	37 (3)	0.792
Prealbumin, g/L	0.200–0.400	0.223 (0.173)	0.262 (0.051)	0.268 (0.041)	0.257 (0.062)	0.316
C-reactive protein, mg/L	< 5.0	3.2 (86.3)	3.1 (0.3)	3.1 (0.0)	3.1 (1.9)	0.423
Interleukin 6, pg./mL	< 10.0	2.0 (11.5)	1.5 (2.7)	1.8 (1.7)	1.8 (3.4)	0.362
Interleukin 1 beta, pg./mL	< 5.0	2.0 (2.1)	2.0 (1.5)	3.9 (3.9)	2.4 (2.1)	0.285
Tumor necrosis factor alpha, pg./mL	< 8.1	8.3 (7.8)	8.4 (5.1)	7.8 (4.8)	6.3 (3.4)	0.740
HbA1c, %	< 6.5	6.3 (3.9)	6.1 (2.6)	6.2 (1.3)	6.4 (2.0)	0.581
Insulin, μIU/mL	6.2–26.1	5.9 (5.6)	6.7 (5.3)	6.9 (6.9)	6.1 (5.2)	0.932
Insulin-like growth factor 1, ng/mL	14.0–647.0	101.2 (116.2)	131.7 (35.8)	141.9 (64.1)^#^^,^^†^	151.9 (61.3)^#^^,^^†^	**0.040**
Triglycerides, mmol/L	0.00–1.90	1.53 (1.49)	1.64 (0.86)	1.44 (1.19)	1.40 (1.06)	0.769
Total cholesterol, mmol/L	< 6.0	4.8 (2.0)	5.0 (0.8)	5.5 (2.1)	5.4 (1.3)	0.460
LDL cholesterol, mmol/L	0.00–3.34	3.09 (1.21)	2.84 (1.05)	2.97 (1.37)	3.01 (1.16)	0.482
HDL cholesterol, mmol/L	> 1.03	1.11 (0.89)	1.82 (1.05)	1.91 (1.17)^#^	1.65 (1.16)^‡^	**0.045**

All data is presented as median (IQR).

1 Changes over time were tested using Friedman test.

# Indicates significant difference from Day 0 based on Conover post-hoc test with correction for false detection rate, *p* < 0.05.

† Indicates significant difference from Day 28 based on Conover post-hoc test with correction for false detection rate, *p* < 0.05.

‡ Indicates significant difference from Day 90 based on Conover post-hoc test with correction for false detection rate, *p* < 0.05.Bold values denote statistical significance at the *p* < 0.05 level.

**Figure 6:**
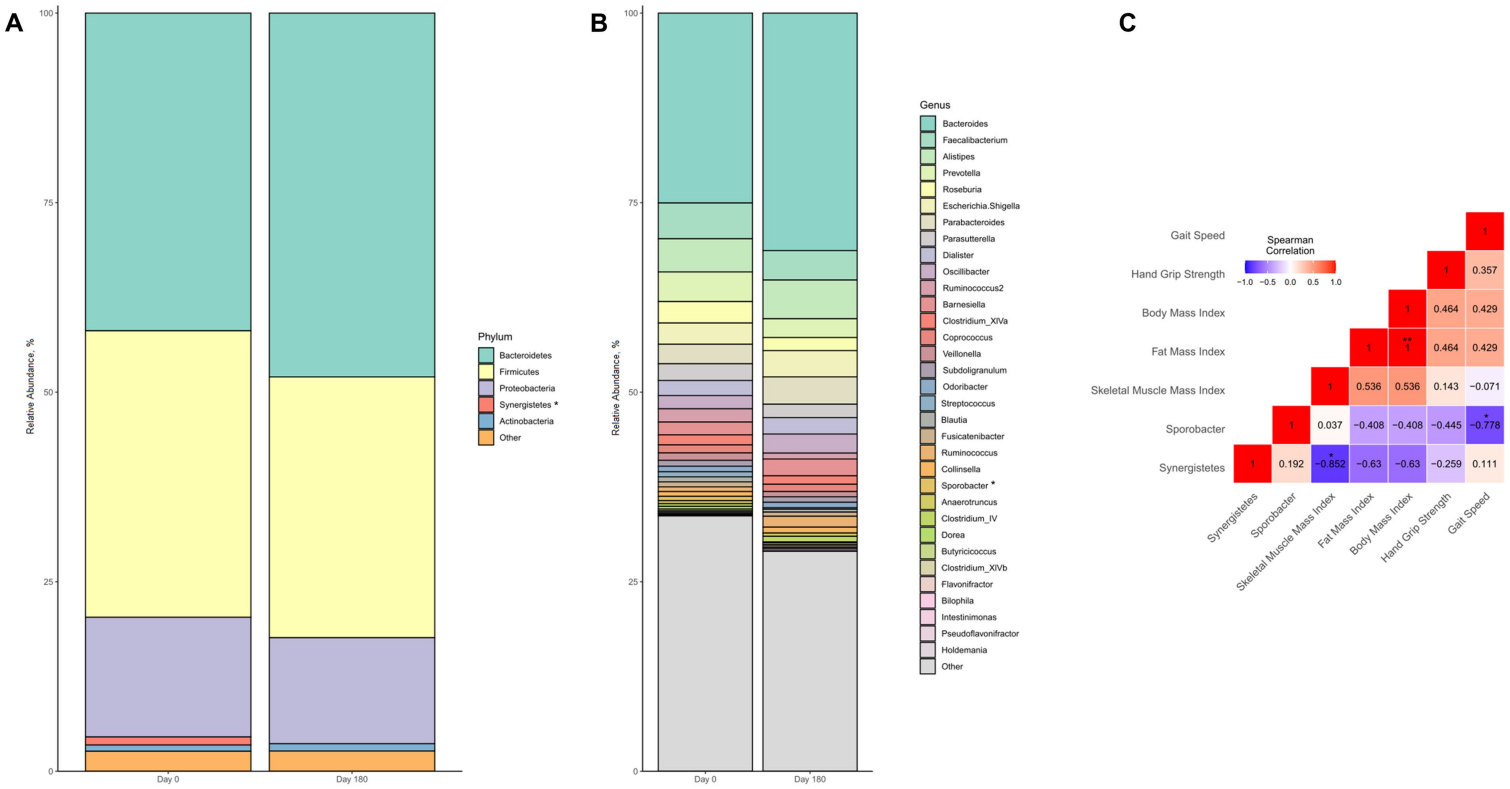
Changes in gut microbiota composition of malnourished patients with chronic pancreatitis in the course of the intensified trans-sectoral nutritional intervention at phyla **(A)** and genera **(B)** level. Correlation between changes in altered gut microbiota taxa and parameters of body composition and muscle function **(C)** (*n* = 7). *p < 0.05; **p < 0.01.

Regarding the intestinal microbiome, we observed no major changes in gut microbiota composition following the intervention (Figures 6A,B). However, there were significant changes in relative abundance of two single taxonomic units, i.e., the phylum *Synergistetes* and the genus *Sporobacter*. Both taxa showed low initial relative abundance of 1.06% (*Synergistetes*) and 0.57% (*Sporobacter*), which significantly decreased even further during the intervention (*p* = 0.046 for each taxa, respectively). Notably, the relative decline in abundance of *Synergistetes* was significantly correlated with gains in skeletal muscle (rho = −0.852, *p* = 0.015) but not weight, fat mass, or parameters of muscle function (Figure 6C). On the other hand, reduction in *Sporobacter* was exclusively associated with improved gait speed (rho = −0.778, *p* = 0.039) but not handgrip strength, body weight or composition (Figure 6C).

## 4 Discussion

Here, we show that a 6-months intensified trans-sectoral nutritional intervention in malnourished patients with chronic pancreatitis not only successfully improves nutritional status but also parameters of disease severity. Moreover, we identify biochemical indicators of ameliorated nutritional status that could serve as future biomarkers.

Our work provides novel insights regarding feasibility of an intensified nutritional invention in patients with chronic pancreatitis. In that regard, there are two particularly noteworthy observations. On the one hand, it must be noted that a substantial fraction of the patients initially screened declined to participate despite being eligible. Although this a common phenomenon in clinical trials, known as Lasagna’s Law (17), in the context of a feasibility study this finding raises questions regarding the reasons for such low participation rate. Because patients by study design were not required to provide reasons for their decision to participate or not, we cannot definitely answer these questions. However, our data shows that especially subjects with severe malnutrition and ongoing pancreatic enzyme replacement therapy participated in this intervention. This could reflect patients’ hopes for improved nutritional status in those, where other modes of therapy have been unsuccessful so far. By contrast, persons with moderate malnutrition may be less motivated to participate because they perceive less impairment due to their compromised nutritional status. Notably, alcoholic etiology or continued substance abuse do not seem to present general barriers for enrollment based on our results. This finding is relevant because alcohol and nicotine abuse are commonly seen in persons with chronic pancreatitis and previous studies have suggested that this could hinder enrollment or retention of patients (18, 19). The second important observation on feasibility is that overall patient adherence was good and the drop-out rate during the intervention was low. An earlier study by Singh et al. (20), testing nutritional interventions for the treatment of malnutrition in chronic pancreatitis, reported similar drop-out rates and adherence to oral nutritional supplementation as well as dietary counseling. Our own previous works suggest that malnourished patients with chronic pancreatitis are susceptible to dietary counseling but supplementation with ONS may be required to reverse malnutrition as shown in the current study (21). Further, it should be considered that the study by Singh and colleagues (20) only lasted 3 months and primarily focused on a dietary intervention, as opposed to the multimodal approach followed in our own investigation. In that respect, it should be noted that behavior regarding alcohol consumption and smoking remained unchanged in most patients. Although abstinence should undisputably be promoted as both alcohol and nicotine abuse are known drivers of disease progression, our results suggest that smoking cessation may not be necessary for improvement of nutritional status. By contrast, inclusion of exercise in the multimodal approach may be a decisive factor in terms of improving muscle mass and function, which in recent years has become a primary objective in the treatment of disease-related malnutrition (22, 23). In our investigation, we observed that patients were highly compliant regarding the exercise component of the intervention. High adherence was likely facilitated by the individual approach targeted to the patients’ performance status. Currently, there is no evidence that supports any specific intervention in terms of physical activity in patients with chronic pancreatitis (24). Although it can be assumed that malnourished patients with chronic pancreatitis will especially benefit from resistance training in terms of reversing muscle loss and inducing protein synthesis (22), more complex and standardized interventions could compromise feasibility. This aspect should be considered as our results show that in general multimodal nutrition therapy in malnourished patients with chronic pancreatitis for up to 6 months is feasible.

In addition to feasibility, we show that this intensified nutritional intervention was highly effective in improving patients’ nutrition status. Previous evidence supporting the efficacy of nutrition therapy in malnourished patients with chronic pancreatitis has been limited to less than a handful of studies (20, 25, 26). Despite heterogeneous study designs and populations these previous investigations consistently suggest that malnutrition can be effectively treated. Besides supporting these earlier findings, our study adds to existing knowledge in several respects. First, we showed that patients not only gained body weight but also muscle mass during the intervention. Second, we also found increased gait speed, which suggests improved muscle performance. Interestingly, hand grip strength remained unchanged. However, this does not necessarily confute an amelioration in muscle function as pathologically reduced muscle strength is rarely seen in malnourished patients with chronic pancreatitis despite low muscle mass (6). Finally, for the first time we report data that suggests that nutritional treatment could lower parameters of overall disease severity and thus may enhance prognosis in chronic pancreatitis. These findings are of high clinical relevance because, although malnutrition is known to be a common complication linked to adverse outcome, definite benefits of dietary interventions in that regard have not been demonstrated. Hence, our findings highlight the potential of nutrition therapy in the treatment of an incurable disease.

Last, in our work we identified multiple biochemical parameters that were associated with changes in nutritional status and could therefore potentially serve as future biomarkers. As for blood parameters, cholinesterase and IGF-1 were significantly associated with improved nutritional status. Cholinesterase, a negative acute phase reactant, has previously been suggested as a suitable indicator of nutritional status and to reflect effectiveness of nutritional support (27). In a previous investigation, we already found cholinesterase to be associated with malnutrition in patients with chronic pancreatitis (6). Cholinesterase may have the advantage of a longer half-life of approximately 12 days (27) over other negative acute phase proteins. Albumin, for instance, has repeatedly been shown to inaccurately reflect nutritional status in case of acute inflammation (28). Notably, in our study, in contrast to cholinesterase neither albumin nor prealbumin changed during the intervention, which supports that these parameters are not suitable to monitor nutritional status in patients with chronic pancreatitis. However, as cholinesterase is also synthesized in the liver, its predictive value for nutritional status could be limited in patients with concomitant liver disease, which is common in patients with alcoholic chronic pancreatitis. Thus, further validation of cholinesterase in a larger study population is needed to be established as a biomarker of nutritional status or efficacy of nutritional support in patients with chronic pancreatitis. As a second blood marker, we identified IGF-1 to be associated with improved nutritional status. As IGF-1 is known to be a key factor regulating protein synthesis in skeletal muscle (29), the increased concentrations seen during the intervention likely reflect enhanced muscle anabolism in patients. Notably, both IGF-1 and skeletal muscle did not further increase between Day 90 and Day 180, which could imply lowered anabolic competence with longer intervention duration (30–32). These observations further support IGF-1 as a promising indicator of muscle anabolism in malnourished patients with chronic pancreatitis. Yet, also in the case of IGF-1, further validation is warranted as its concentrations are correlated with various other factors, for instance, dietary protein intake and physical activity (33, 34). Besides blood markers, we also detected changes in gut microbiota composition to be associated with improved nutritional status. Stool-based biomarkers offer the advantage of being non-invasive and could overcome some of the shortcomings of blood markers. We found only two taxa that both significantly declined in abundance during the intervention. Both the genus *Sporobacter* and the phylum *Synergistetes* have been linked to pathologically reduced muscle mass and function before (35, 36). Increased *Sporobacter* abundance has been observed in patients with amyotrophic lateral sclerosis, a neurodegenerative disease accompanied by progressive muscular atrophy (35). In line with this, we found a reduction in abundance to be associated with increased skeletal muscle mass in our patients. Further, Peng et al. reported higher abundance of *Synergistetes* in sarcopenic patients with heart failure than in non-sarcopenic controls (36). Interestingly, Peng et al. also considered lowered gait speed as a diagnostic criterion for sarcopenia in their study, which corroborates our observation of an inverse correlation between changes in relative abundance of *Synergistetes* and gait speed. However, our results should be interpreted with caution. For one thing, chronic pancreatitis itself is associated with a distinct gut microbiota composition compared to healthy controls (37) and secondly the multimodal intervention addressed multiple factors, including diet, physical activity, and smoking, which all evidently exert an effect on microbiota composition (38). Therefore, our findings, for now, should be considered explorative with the need for validation in future investigations testing the usability of gut microbiota composition as a nutritional marker in chronic pancreatitis.

In conclusion, we demonstrate that malnourished patients with chronic pancreatitis benefit from an intensified nutritional treatment. Benefits exceed improved nutritional status by also reducing parameters of disease severity and thus potentially prognosis, which highlights that nutrition therapy is a powerful asset in the management of this progressive and incurable disease. Implementation of an intensified trans-sectoral intervention in this patient group seems feasible but superiority to standard-of-care treatment needs to be shown in an adequately powered randomized controlled trial. Likewise, potential biomarkers for assessment of nutritional status and efficacy of nutritional intervention require further validation.

## Data Availability

The datasets presented in this article are not readily available because of ethical and legal considerations. Requests to access the datasets should be directed to the corresponding author.
