# Supplementary material for: An intensified trans-sectoral nutritional intervention in malnourished patients with chronic pancreatitis improves diseases prognosis and identifies potential biomarkers of nutritional status
Source: Front Med (Lausanne). 2024 Oct 8;11:1446699. doi: 10.3389/fmed.2024.1446699 (PMC11493651; doi:10.3389/fmed.2024.1446699)
Supplement: Supplementary file 1 [file Table_1.DOCX]

Supplementary Table 1 Changes in intakes of energy and macronutrients, excluding oral nutritional supplements, of malnourished patients with chronic pancreatitis in the course of the intensified trans-sectoral nutritional intervention (n=9).

|  | **Day 0** | **Day 28** | **Day 90** | **Day 180** | p-value^1^ |
| --- | --- | --- | --- | --- | --- |
| Energy, kcal/d | 2244 (1380) | 1773 (1059) | 2240 (1545) | 2447 (828) | .737 |
| Energy, kcal/kg body weight/d | 41 (28) | 30 (24) | 35 (26) | 37 (17) | .833 |
| Protein, g/d | 83 (52) | 69 (56) | 83 (57) | 90 (36) | .204 |
| Protein, g/kg body weight/d | 1.4 (0.9) | 1.1 (0.9) | 1.2 (0.8) | 1.4 (0.7) | .506 |
| Carbohydrates, g/d | 266 (216) | 241 (128) | 292 (169) | 244 (51) | .865 |
| Fat, g/d | 91 (54) | 61 (51) | 98 (82) | 88 (62) | .091 |
| Dietary fiber, g/d | 20 (15) | 20 (9) | 17 (9) | 21 (14) | .971 |
| Alcohol, g/d | 0 (2) | 0 (1) | 0 (2) | 0 (1) | .564 |

All data is presented as median (IQR)

^1^ Changes over time were tested using Friedman test
