# Supplementary material for: An intensified trans-sectoral nutritional intervention in malnourished patients with chronic pancreatitis improves diseases prognosis and identifies potential biomarkers of nutritional status
Source: Front Med (Lausanne). 2024 Oct 8;11:1446699. doi: 10.3389/fmed.2024.1446699 (PMC11493651; doi:10.3389/fmed.2024.1446699)
Supplement: Supplementary file 2 [file Table_2.DOCX]

Supplementary Table 2 Changes in physical activity of malnourished patients with chronic pancreatitis in the course of the intensified trans-sectoral nutritional intervention (n=9).

|  | **Day 0** | **Day 28** | **Day 90** | **Day 180** | p-value^1^ |
| --- | --- | --- | --- | --- | --- |
| **Physical activity level, n (%)** |  | ^#^ | ^#^ | ^#^ | 0.012 |
| Low | 2 (22) | 0 (0) | 0 (0) | 0 (0) |  |
| Moderate | 2 (22) | 2 (22) | 1 (11) | 2 (22) |  |
| High | 5 (56) | 7 (78) | 8 (89) | 7 (78) |  |

^1^ Changes over time were tested using Friedman test considering physical activity level as an ordinal variable

^#^ Indicates significant difference from Day 0 based on Conover post-hoc test with correction for false detection rate, p <0.001
