# Supplementary material for: An intensified trans-sectoral nutritional intervention in malnourished patients with chronic pancreatitis improves diseases prognosis and identifies potential biomarkers of nutritional status
Source: Front Med (Lausanne). 2024 Oct 8;11:1446699. doi: 10.3389/fmed.2024.1446699 (PMC11493651; doi:10.3389/fmed.2024.1446699)
Supplement: Supplementary file 3 [file Table_3.DOCX]

Supplementary Table 3 Changes in scoring of individual parameters included in Chronic Pancreatitis Prognosis Score (COPPS) of malnourished patients with chronic pancreatitis in the course of the intensified trans-sectoral nutritional intervention (n=9).

|  | **Day 0** | **Day 28** | **Day 90** | **Day 180** | p-value^1^ |
| --- | --- | --- | --- | --- | --- |
| **Parameter, pts.** |  |  |  |  |  |
| Numeric rating scale of pain | 1 (1) | 1 (1) | 1 (1) | 1 (1) | 0.908 |
| HbA1 | 1 (1) | 1 (2) | 1 (1) | 1 (1) | 0.145 |
| C-reactive protein | 2 (2) | 1 (1) | 1 (0) | 1 (1) | 0.068 |
| Body mass index | 2 (0) | 2 (0) | 2 (1)^#^ | 2 (1)^#†^ | **0.036** |
| Thrombocytes | 1 (2) | 1 (2) | 1 (0) | 1 (0) | 0.061 |

For each parameter 1, 2, or 3 points were assigned as described by Beyer et al. (13). COPPS A= 5-6 points; COPPS A= 7-9 points; COPPS A= 10-15 points

All data is presented as median (IQR)

^1^ Changes over time were tested using Friedman test

^#^ Indicates significant difference from Day 0 based on Conover post-hoc test with correction for false detection rate, p < 0.01

^†^ Indicates significant difference from Day 28 based on Conover post-hoc test with correction for false detection rate, p < 0.01
